# Worldwide Dissemination of the NDM-Type Carbapenemases in Gram-Negative Bacteria

**DOI:** 10.1155/2014/249856

**Published:** 2014-03-26

**Authors:** Laurent Dortet, Laurent Poirel, Patrice Nordmann

**Affiliations:** ^1^INSERM U914 “Emerging Resistance to Antibiotics”, 78 Avenue du Général Leclerc, 94270 Le Kremlin-Bicêtre, France; ^2^Medical and Molecular Microbiology Unit, Department of Medicine, Faculty of Science, University of Fribourg, 3 Rue Albert Gockel, 1700 Fribourg, Switzerland

## Abstract

The emergence of one of the most recently described carbapenemases, namely, the New Delhi metallo-lactamase (NDM-1), constitutes a critical and growingly important medical issue. This resistance trait compromises the efficacy of almost all lactams (except aztreonam), including the last resort carbapenems. Therapeutical options may remain limited mostly to colistin, tigecycline, and fosfomycin. The main known reservoir of NDM producers is the Indian subcontinent whereas a secondary reservoir seems to have established the Balkans regions and the Middle East. Although the spread of *bla*_NDM_-like genes (several variants) is derived mostly by conjugative plasmids in Enterobacteriaceae, this carbapenemase has also been identified in *P. aeruginosa* and *Acinetobacter* spp. *Acinetobacter* sp. may play a pivotal role for spreading *bla*_NDM_ genes for its natural reservoir to Enterobacteriaceae. Rapid diagnostic techniques (Carba NP test) and screening of carriers are the cornerstone to try to contain this outbreak which threatens the efficacy of the modern medicine.

## 1. Introduction

During the last decade the emergence of carbapenemase-producing strains among Enterobacteriaceae,* Pseudomonas* spp., and* Acinetobacter baumannii* is remarkable. A variety of carbapenemases have been reported such as the Ambler class A KPC-type (mostly identified in Enterobacteriaceae and* Pseudomonas aeruginosa*) and GES-type (mostly in* A. baumannii*), the Ambler class B metallo-*β*-lactamases (MBL) of VIM-, IMP-, GIM-, and NDM-types, and the Ambler class D carbapenemases of the OXA-48 type in Enterobacteriaceae and of OXA-23, OXA-24/-40, OXA-58, and OXA-143 types in* Acinetobacter* spp. The emergence of the most recently described carbapenemase, namely, the New Delhi metallo-*β*-lactamase (NDM-1), constitutes a critical medical issue. Indeed, this enzyme compromises the efficacy of almost all *β*-lactams (except aztreonam), including the last resort carbapenems. Although most of the NDM-producing strains identified are Enterobacteriaceae, this carbapenemase has also been reported from* Acinetobacter* spp. and more rarely from* P. aeruginosa*, both species causing severe nosocomial infections, including urinary tract infections, peritonitis, septicemia, and pulmonary infections. The Indian subcontinent, the Balkans regions, and the Middle East are considered to be the main reservoirs of NDM producers. Since therapeutical options are limited to very few antibiotics such as colistin, tigecycline, and fosfomycin, hospital- and community-acquired infections caused by NDM-1 producers are difficult to eradicate. Isolation of infected patients and carriers and rapid diagnostic techniques are the key factors that contribute to contain this outbreak that threatens the efficacy of the modern medicine.

## 2. Clinical Impact of the Antibiotic Resistance Patterns of NDM Producers for the Treatment

Currently, one of the most clinically significant carbapenemase is the recently described NDM-1 (New Delhi metallo-*β*-lactamase). This carbapenemase belongs to the class B of Ambler *β*-lactamases classification that includes the metallo-*β*-lactamases (MBLs). NDM-1 shares very little identity with other MBLs, the most similar being VIM-1/VIM-2 with only 32.4% amino acid identity. Compared to VIM-2, NDM-1 displays tighter binding to most cephalosporins, in particular to cefuroxime (*K*_mNDM-1_ = 8 *μ*M, *K*_mVIM-2_ = 22 *μ*M), cefotaxime (*K*_mNDM-1_ = 10 *μ*M, *K*_mVIM-2_ = 32 *μ*M), cephalothin (*K*_mNDM-1_ = 10 *μ*M, *K*_mVIM-2_ = 44 *μ*M), and penicillins (*K*_mNDM-1_ = 16 *μ*M, *K*_mVIM-2_ = 49 *μ*M). Like all other MBLs, NDM-1 efficiently hydrolyses a broad range of *β*-lactams including penicillins, cephalosporins, and carbapenems, just sparing monobactams such as aztreonam. NDM-1 does not bind to carbapenems as tightly as IMP-1 or VIM-2 does, and the turnover rate of carbapenem hydrolysis is similar to that of VIM-2 (*k*_cat_/*K*_m_ are 0.21, 1.2, and 0.99 s^−1^·*μ*M^−1^ for NDM-1, IMP-1, and VIM-2, resp.). Similar to the other MBLs, the active site of NDM-1 contains two metal ion binding sites: the His and Cys sites. Accordingly, a 3D-structure modelling of the NDM-1 enzyme showed that two zinc ions were present at both the His and Cys sites with a distance of 4.20 Å [1]. Indeed, the hydrolysis activity of MBLs depends on the interaction of the *β*-lactam molecule with Zn^2+^ ion(s) in their active site. Consequently, their activity is inhibited by chelators of divalent cations, such as EDTA. Accordingly, the efficacy of EDTA (Ca-EDTA) has been evaluated in a mouse model of sepsis caused by an NDM-1-producing* Escherichia coli*. It has been shown that a combination therapy using imipenem/cilastatin sodium (IPM/CS) and Ca-EDTA reduced the bacterial inoculum, as compared to IPM/CS alone suggesting the possibility to use Ca-EDTA in clinical therapeutics [2]. Comparison of IMP-1, VIM-2, and NDM-1 by an* in silico* approach revealed that NDM-1 might have greater drug profile and catalytic efficiency than IMP-1 and VIM-2 due to a larger pocket opening and a lower distance between the Zn-I ion and *β*-lactam oxygen of the carbapenem [3].

**Figure 1:**
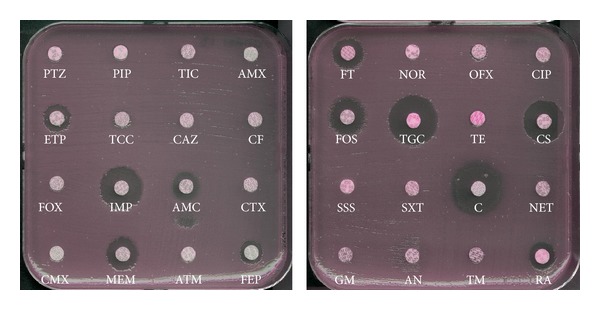
Antibiogram of a NDM-1-producing* K. pneumoniae* isolate. The *bla*_NDM-1_ gene was located onto a IncHIIB plasmid of ca. ~200 kb in that strain that also harbored two additional *β*-lactamase genes (*bla*_CTX-M-15_, *bla*_SHV-12_, *bla*_OXA-1_) and an aminoglycoside methylase (*armA*) responsible for high-level resistance to all aminoglycosides. PTZ, piperacillin + tazobactam; PIP, piperacillin; TIC, ticarcillin; AMX, amoxicillin; ETP, ertapenem; TCC, ticarcillin + clavulanic acid; CAZ, ceftazidime; CF, cefalotin; FOX, cefoxitin; IMP, imipenem; AMC, amoxicillin + clavulanic acid; CTX, cefotaxime; CMX, cefuroxime; MEM, meropenem; ATM, aztreonam; FEP, cefepime; FT, nitrofurantoin; NOR, norfloxacin; OFX, ofloxacin; CIP, ciprofloxacin; FOS, fosfomycin; TGC, tigecycline; TE, tetracycline; CS, colistin; SSS, sulfonamide; SXT, sulfamethoxazole + trimethoprim; C, chloramphenicol; NET, netilmicin; GM, gentamicin; AN, amikacin; TM, tobramycin; RA, rifampicin.

It is noteworthy that a quite systematic association with other antibiotic resistance determinants is observed in almost all NDM producers (Enterobacteriaceae,* Acinetobacter,* and* Pseudomonas*). Those associated resistance determinants are AmpC cephalosporinases, clavulanic acid inhibited expanded-spectrum *β*-lactamases (ESBLs), other types of carbapenemases (OXA-48-, VIM-, and KPC-types), and resistance to aminoglycosides (16S RNA methylases), to quinolones (Qnr), to macrolides (esterases), to rifampicin (rifampicin-modifying enzymes), to chloramphenicol, and to sulfamethoxazole [4–9]. Consequently, most of the NDM-1 producers remain susceptible only to two bactericidal antibiotics (colistin and fosfomycin) and a single bacteriostatic antibiotic (tigecycline) [10, 11] (Figure 1).* In vitro* synergy combination assays performed with NDM-1 producers with those three antibiotic molecules showed a synergistic activity of colistin and fosfomycin, of colistin and tigecycline in rare cases, whereas most of the antibiotic associations remain neutral for most of the tested isolates [12]. Since NDM-1 does not hydrolyze aztreonam, a combination therapy including aztreonam and avibactam (also named NXL-104), a novel serine *β*-lactamase inhibitor inhibiting the most frequent broad-spectrum hydrolyzing-*β*-lactamases hydrolyzing aztreonam has been suggested as a possible strategy against NDM-1-producing Enterobacteriaceae. This therapeutic option seems to be a very efficient combination therapy* in vitro *[13, 14].

## 3. Infections Caused by NDM Producers

Since NDM producers were mainly described in Enterobacteriaceae, infections caused by NDM producers include urinary tract infections, peritonitis, septicemia, pulmonary infections, soft tissue infections, and device-associated infections. As observed for other multidrug-resistant bacteria, it is highly probable that colonization of the gut flora might precede the infection by NDM producers and orofecal transmission in the community might occur mostly through hand contamination, food, and water. Among the NDM-1-producing Enterobacteriaceae,* Klebsiella pneumoniae* and* E. coli* are the most often described species. Both hospital- and community-acquired infections have been reported. However, this carbapenemase is also frequently described in other enterobacterial species including* Klebsiella oxytoca*,* Enterobacter cloacae*,* Citrobacter freundii*,* Proteus mirabilis*,* Salmonella* spp., and* Providencia* spp. Although most of NDM-producing bacteria are Enterobacteriaceae, this carbapenemase was also reported from* Acinetobacter* spp. [15–32] and in rare cases* Pseudomonas aeruginosa* [33, 34].

Since, no specific virulence factor is known to be associated with *bla*_NDM-1_-carrying plasmids [6, 35–39], there is no evidence that NDM-producing bacteria are more virulent than other strains [40–42]. However, some rare isolates of NDM-1-producing virulent enteric bacteria such as* Salmonella* [43–45] and* Vibrio cholerae *[46, 47] have been described.

## 4. Epidemiology of NDM-Producing Bacteria

**Figure 2:**
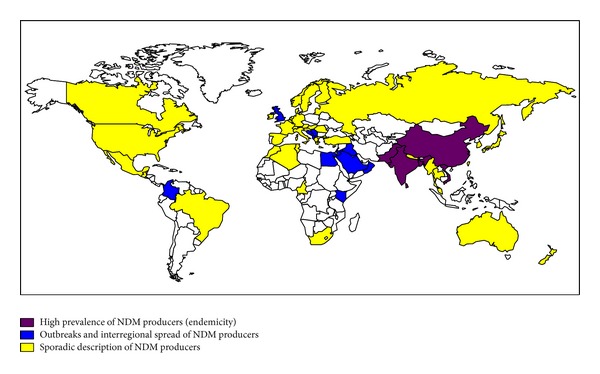
Geographical distribution of NDM producers.

NDM-1 was first identified in 2008 in a* K. pneumoniae* isolate recovered from a Swedish patient who has been previously hospitalized in New Delhi, India [48]. Since then, NDM carbapenemases are the focus of worldwide attention due to the rapid dissemination of the corresponding gene among Enterobacteriaceae and* Acinetobacter* spp. mainly (Figure 2). Rapidly, a link between NDM-producing Enterobacteriaceae and the Indian subcontinent has been pointed out [49–51], and prevalence rates of NDM-producing Enterobacteriaceae were found to range from 5 to 18.5% in Indian and Pakistan hospitals [52–55]. In addition, the *bla*_NDM-1_ gene was detected not only in patient samples, but also in drinking water and seepage samples in New Delhi [47]. The occurrence of NDM-1-producing bacteria in environmental samples in New Delhi is significant for people living in the city who often rely onto public water and poor sanitation facilities. A secondary reservoir of NDM-1 producers was then highlighted through several studies reporting patients colonized or infected with NDM-1 producers originating from the Balkan states [50, 56–61]. Recent reports also suggested that the Middle East might be an additional reservoir of NDM producers [62–67]. This dissemination of NDM producers in the Middle East could mostly be linked to the population exchange between the Middle East and the Indian subcontinent. However, NDM-1 producing bacteria have now been reported worldwide with a rapid dissemination from the two previously described reservoirs, namely, the Indian subcontinent and the Balkan countries.

As observed with the dissemination of NDM-1-producing Enterobacteriaceae, NDM-producing* Acinetobacter* has also been recovered from environmental samples in China [31]. Currently, the majority of NDM-producing* Acinetobacter* spp. are reported from China [18, 19, 25, 29–32] and Middle East [17, 20–24, 26].

## 5. Genetic Features of the *bla*_NDM_ Genes

**Figure 3:**
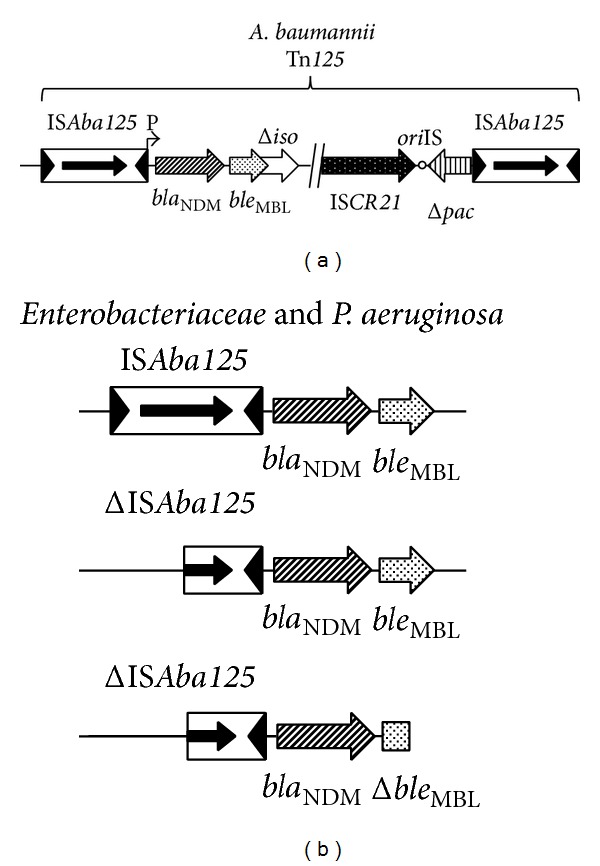
Schematic representation of *bla*_NDM_-associated genetic structures identified among Gram-negative clinical isolates. (a) Structure found in* A. baumannii*, where the *bla*_NDM_ gene is part of the composite transposon Tn*125.* (b) Structures found in Enterobacteriaceae and* P. aeruginosa* where IS*Aba125* is presented as full or truncated element with *ble*_MBL_ gene (bleomycin resistance encoding gene) also being present as full or truncated gene. Genes and their corresponding transcription orientations are represented by horizontal arrows.* ori*IS of IS*CR21 *is indicated by a circle. The *bla*_NDM_ promoter is indicated (P). IS, insertion sequence; gene names are abbreviated according to their corresponding proteins: *ble*_MBL_, bleomycin resistance gene; Δ*iso* for truncated phosphoribosylanthranilate isomerase; Δ*pac* for truncated phospholipid acetyltransferase.

In Enterobacteriaceae, the *bla*_NDM-1_ gene is located mostly onto conjugative plasmids belonging to several incompatibility groups [6, 7, 25, 35, 37, 38, 63, 68]. However, investigation of a worldwide collectionof NDM-1-producing enterobacterial isolates showed that the current spread of the *bla*_NDM-1_ gene is not related to the spread of specific clones, specific plasmids, or single genetic structure [7]. In* Acinetobacter* spp. the *bla*_NDM_-type genes are found to be either plasmid- or chromosome-located, and in the rare NDM-1-producing* P. aeruginosa*, the *bla*_NDM-1_ gene was found to be chromosomally located [33, 34]. Investigations on the immediate genetic environment of *bla*_NDM_ genes revealed the presence of a conserved structure that always associated the complete or truncated insertion sequence IS*Aba125 *at the 5′-end and the *ble*_MBL_ gene (encoding resistance to the anticancer drug bleomycin) at the 3′-end of the *bla*_NDM_ genes [69] (Figure 3). In addition, in several studies focusing on NDM-producing* A. baumannii*, the *bla*_NDM_ gene was located between two copies of the IS*Aba125* element, forming a composite transposon named Tn*125* [15–17, 20, 24, 26, 70, 71] (Figure 3). Systematic identification of a truncated form of this composite transposon in Enterobacteriaceae, while it was described in its entire form in* A. baumannii*, strongly suggesting that* Acinetobacter* spp. has been a reservoir of those *bla*_NDM_ genes before targeting enterobacterial species. Those findings highlight that even though* A. baumannii* is usually recognized as a final acceptor for resistance genes, it may acquire several resistance determinants and then transfer them to Enterobacteriaceae and* Pseudomonas* spp.

## 6. NDM Variants

**Figure 4:**
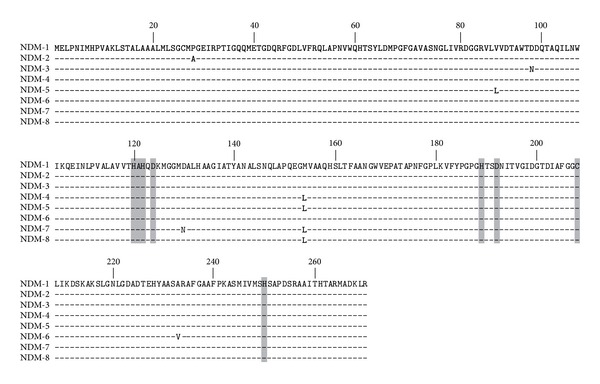
Alignment of the amino acid sequences of the eight reported NDM variants. Conserved residues of the active site of metallo-*β*-lactamases are highlighted in gray. The bolded leucine in position 154 has been described to be responsible to an increased carbapenem hydrolysis.

Since the first description of NDM-1, eight variants of this enzyme have been published (NDM-1 to -8) (Figure 4) and ten have been assigned (http://www.lahey.org). The first variant NDM-2 is a point mutation variant having a C to G substitution at position 82 resulting in an amino acid substitution of a proline to an alanine residue at position 28 (Pro → Ala) (Figure 4) [26]. Considering that this point mutation was located at the last amino acid of the peptide leader of the enzyme, MIC values of *β*-lactams including carbapenems showed no significant difference between NDM-1 and NDM-2 producers. NDM-2 has been identified in several* A. baumannii* strains [20–22, 26] but not yet in Enterobacteriaceae. The NDM-3 variant was described from an* E. coli* isolate and differs from NDM-1 by a single nucleotide change conferring a peptide sequence change at position 95 (Asp → Asn) that does not modify the hydrolytic activities of the enzyme (Figure 4) [11]. The NDM-4 variant differs from NDM-1 by a single amino acid substitution at position 154 (Met → Leu) (Figure 4). Kinetic data showed that this amino acid substitution is responsible for an increased hydrolytic activity of NDM-4 compared to NDM-1 toward cefalotin, ceftazidime, cefotaxime, imipenem, and meropenem, whereas cefepime was less hydrolyzed [72]. The NDM-5 variant shares the substitution at positions 154 (Met → Leu) with NDM-4, conferring enhanced hydrolytic activity against carbapenems and harbors a second amino acid substitution at position 88 (Val → Leu) (Figure 4) [73]. The NDM-6 variant differs from NDM-1 by a single amino acid substitution at position 233 (Ala → Val) leading to no obvious modification in the hydrolytic activity of the enzyme (Figure 4) [74]. The NDM-7 variant was concomitantly described from an* E. coli* isolate recovered from a French patient who had travelled to Burma [75] and an* E. coli* isolate recovered from a Yemeni patient previously hospitalized at the Frankfurt University Hospital in Germany [76]. The *bla*_NDM-7_ gene differs from *bla*_NDM-1_ by two amino-acid substitutions at positions 388 (G→A) and 460 (A→C) corresponding to amino acid substitutions at position 130 (Asp → Asn) and 154 (Met → Leu), respectively (Figure 4). The amino acid substitution at position 154 (Met → Leu) increases the hydrolysis activity of the enzyme [75, 76]. The amino acid sequence of the last published NDM variant, namely, NDM-8, has substitutions at positions 130 (Asp → Gly) and 154 (Met → Leu) compared with NDM-1 (Figure 4) [77]. This NDM variant possesses the amino acid substitution at position 154 (Met → Leu), but its critical impact on *β*-lactams hydrolysis has not been detailed.

## 7. Identification of NDM Producers

Detection of carbapenemase producers, including NDM producers, in clinical specimens is based currently on a preliminary analysis of susceptibility testing results. The US guidelines (CLSI) (updated in 2013) retained as breakpoints for Enterobacteriaceae susceptibility (*S*) ≤ 1 and resistance (*R*) > 4 mg/L for imipenem and meropenem and *S* ≤ 0.5 and *R* > 2 mg/L for ertapenem. The European guidelines (EUCAST) (updated in 2013) are slightly different and propose breakpoints for imipenem and meropenem as follows: susceptible (*S*) ≤ 2 and resistant (*R*) > 8 mg/L and for ertapenem *S* ≤ 0.5 and *R* > 1 mg/L. Although some discrepancies might exist for several isolates depending on the reference used to interpret the antibiogram, MIC values of ertapenem are often higher than those of other carbapenems with NDM producers. Consequently, ertapenem would be the best molecule for suspecting most of the carbapenemase producers, including NDM producers, and constitutes good screening criteria. Notably, this greater sensitivity of ertapenem compared to the other carbapenems is counterbalanced by its lower specificity. Of note, susceptibility to carbapenems is observed for some NDM producers and additional tests for carbapenemase detection are needed to detect them accurately.

### 7.1. Detection of a Carbapenemase Activity

One of the commonly used techniques is the modified Hodge test (MHT), which has been used for years. Unfortunately, the MHT has been proved to lack sensitivity (50%) for detecting NDM-1 producers. Of note, ZnSO_4_ (100 *μ*g/mL) supplementation in the culture medium significantly increases the sensitivity to 85.7% [78]. However, this test has a low specificity with* Enterobacter* spp. often overexpressing their chromosomal cephalosporinase [79]. In addition, results of the MHT are obtained at least 72 h after the bacterial identification.

UV spectrophotometry analysis of carbapenem hydrolysis has been developed to detect carbapenem hydrolysis. This method is based on the detection of the decrease of imipenem absorbance with crude extracts of bacterial enzymes. Crude extracts can be obtained from an overnight culture of the tested strain after mechanical lysis. This UV spectrophotometry-based technique is cheap and has a 100% sensitivity and a 98.5% specificity for detecting carbapenemase activity [80]. However, it is time-consuming and requires trained microbiologists and expensive equipment.

Analysis of carbapenem hydrolysis by using the MALDI-TOF technology has been shown to be a useful technique to detect carbapenemase production in a few hours. This technique was based on detection of a carbapenem (imipenem, meropenem, or ertapenem) spectrum and of its main derivatives resulting from carbapenem hydrolysis. After 3 to 4 hours of incubation of the tested isolate with a carbapenem, the bacteria were pelleted by centrifugation and the supernatant containing the carbapenem and its metabolites was tested by MALDI-TOF mass spectrophotometry. Disappearance of the peak corresponding to the native carbapenem and appearance of peak(s) corresponding to the metabolite(s) resulting on the carbapenem hydrolysis sign a carbapenemase activity [81–85]. This test has excellent sensitivity and specificity. However, it again requires trained microbiologists and expensive equipment.

**Figure 5:**
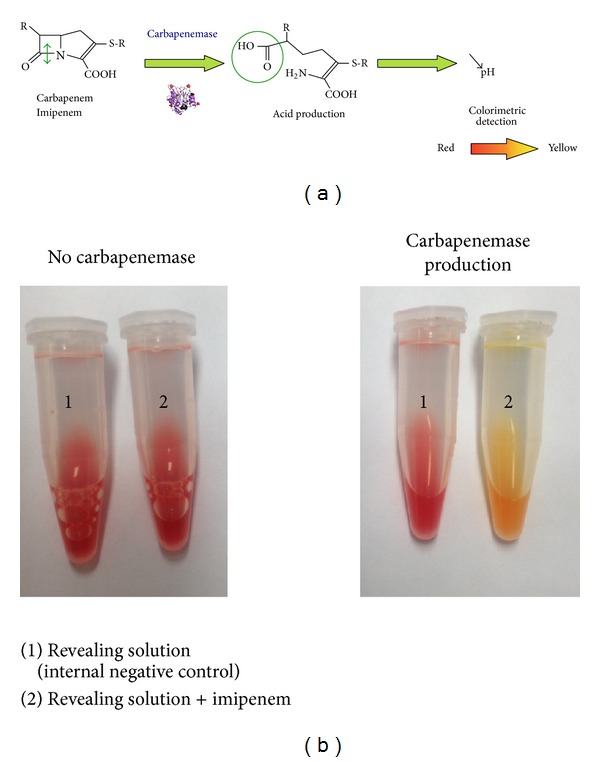
Principle (a) and interpretation (b) of the Carba NP test recently developed for the rapid identification of carbapenemase producers among Enterobacteriaceae and* Pseudomonas* spp.

The most promising technique is the rapid Carba NP test. It is based on the detection of the hydrolysis of imipenem by a color change of a pH indicator (Figure 5). This test is 100% sensitive and 100% specific for the detection of any type of carbapemenase produced by Enterobacteriaceae including NDM producers [86–88]. The Carba NP test has been also validated for the detection of most carbapenemase-producing* Pseudomonas* spp., including all NDM producers [89]. A second version of the Carba NP test (the Carba NP test II) has been developed to rapidly differentiate between the diverse carbapenemase types found in Enterobacteriaceae and* P. aeruginosa*. This Carba NP test II combines the inhibition properties of EDTA with the high efficiency of the Carba NP test for identification of any type of MBL producer, including all NDM producers [90]. Recently, the Carba NP test has been evaluated to detect carbapenemase-producing Enterobacteriaceae (*n* = 193) directly from spiked blood cultures. The proposed strategy allows detection of all NDM producers (*n* = 33) in less than 5 hours, with sensitivity and specificity of 100%, respectively [91]. This test has excellent sensitivity and specificity. However, it requires homemade reagents that are not yet commercially available.

### 7.2. Phenotypic Detection of Metallo-*β*-Lactamase (MBL) Producing Isolates

Detection methods based on the inhibitory properties of several divalent ions chelators (e.g., EDTA and dipicolinic acid) may identify MBL producers. A disk-diffusion test based on the detection of a synergy between a carbapenem-containing disk (imipenem or meropenem) and a disk containing an MBL inhibitor (EDTA or mercaptopropionic acid or dipicolinic acid) has been proposed [92].

A combined disk technique using a carbapenem disk and the same carbapenem disk supplemented with EDTA (10 *μ*L of a 0.1 M solution at pH 8) has been also proposed [93]. Using this test, a 5 mm increase of the inhibition diameter around the disk containing imipenem plus EDTA compared to imipenem alone likely indicates the production of a MBL. However, those two phenotypic methods are time-consuming and false-negative results often arise, in particular when low level of resistance is observed [93].

Among those phenotypic methods, the Etest MBL strip, a two-sided strip containing gradients of imipenem alone on one side and imipenem supplemented with EDTA on the other side, is also commonly used for the detection of MBL producers. Using this test, at least three doubling dilutions of the MIC in the presence of EDTA are considered as a positive result [94]. However, several NDM-producing isolates exhibit low MIC of carbapenems, leading to not interpretable results using the Etest MBL strip.

### 7.3. Molecular Detection of NDM Producers

All the previous techniques can detect the carbapenemase production and, in some cases more precisely, production of an MBL, but none of them is able to specifically identify an NDM enzyme or its corresponding gene. Therefore a number of genotypic approaches have been reported, based on PCR techniques, including real-time PCR methods able to detect *bla*_NDM_-positive isolates directly from clinical samples [95, 96]. Those methods, however, have the disadvantage to be unable to identify any novel carbapenemase gene and are quite expensive. Commercial DNA microarray methods are marketed and increase the convenience of those tests [97]. Although they cannot overcome general limitations of genotypic techniques those DNA microarrays are able to identify the presence of carbapenemase and the main extended-spectrum *β*-lactamase and acquired cephalosporinases genes. Accordingly, this technique is more adapted for an epidemiological purpose in order to control an outbreak. Finally, molecular amplification of the *bla*_NDM_ gene followed by sequencing is needed to identify the exact nature of the NDM variant.

## 8. Detection of Infected and Colonized Patients

Since the prevention of dissemination of carbapenemase producers partially relies on an early and accurate detection of carriers, recommendations for the screening of colonized patients have been introduced in several countries. Commonly, “at-risk” patients, meaning those being colonized with carbapenemase producers, are patients transferred from a foreign hospital and those hospitalized in intensive-care units, in transplantation units, and immunocompromised patients.

Since the intestinal flora is the main reservoir of Enterobacteriaceae, rectal swabs and stools are the most suitable clinical samples for performing this screening. These specimens may be plated on screening medium, either directly or after an enrichment step in broth containing imipenem 0.5–1 *μ*g/mL or ertapenem 0.5 *μ*g/mL [98, 99]. In outbreak situations, this enrichment step might increase the sensitivity of the screening and consequently reduce the number of potential false-negative results by increasing the inoculum of the targeted strain. On the opposite, its disadvantage is the induced delay (12–24 h) needed to confirm or reject carbapenemase detection. Although the efficiency of this enrichment step has not been evaluated for NDM producers, it has already been shown to improve the detection of KPC producers.

Regardless of the enrichment step, the specimens have to be plated on selective media. For that purpose, several screening media have been evaluated and compared to the screening of carriers of NDM producers. One of the first tested medium was the ChromID ESBL culture medium (bioMérieux) containing cefpodoxime used as a selector and which is routinely used to screen ESBL producers. Since NDM enzymes have a broad-spectrum activity, they hydrolyze not only carbapenems but also expanded-spectrum cephalosporins very efficiently. Therefore, detection of NDM-producing isolates using ChromID ESBL (aimed do detect ESBL producers) is possible but with a low specificity since the selective agent is a cephalosporin and not a specific carbapenemase substrate (e.g., a carbapenem). Several media supplemented with a carbapenem have been developed and marketed for the screening of carbapenemase producers. The first screening medium targeting KPC producers was the CHROMagar KPC medium that contains meropenem (CHROMagar, Paris, France) [100]. Using this medium, carbapenem-resistant bacteria are well detected when they exhibit relatively high-level resistance to carbapenems. Its main disadvantage remains in its lack of sensitivity, since it does not detect carbapenemase producers with low-level carbapenem resistance. Indeed, although NDM producers have often high level resistance to carbapenems, several isolates that exhibited MICs comprised between 0.5 and 1 *μ*g/mL, making their detection difficult on screening media containing high concentration of carbapenems [78, 93, 101]. Colorex KPC (E&O laboratories, Bonnybridge, UK), another screening medium for carbapenemase producers, also contains meropenem. Since the content of this medium is reported to be identical to that of CHROMagar KPC, only NDM producers with high-level resistance to carbapenems may be detected, leading to an accurate detection of 57% to 64% of NDM-producing Enterobacteriaceae using this medium [55, 102]. A third commercially available screening medium also contains a carbapenem (CRE Brilliance, Thermo Fisher Scientific, UK). Depending on the study, sensitivities for detection of patients colonized with NDM producers were reported to be 63% to 85% using this medium [102, 103]. Another screening medium also containing a carbapenem is the ChromID CARBA (bioMérieux, La Balmes-les-Grottes, France). This commercially available medium has been reported to be more sensitive (87.5% to 94%) than the others for the detection of NDM-producing Enterobacteriaceae [55, 102, 103]. Finally, a homemade screening medium containing ertapenem, cloxacillin, and zinc, namely, the SUPERCARBA medium, has an excellent sensitivity and specificity for the detection of carbapenemase producers, including NDM producers. The zinc supplementation and the low ertapenem concentration allow the efficient detection of all NDM producers regardless of their level of resistance to carbapenems [101, 104]. Consequently, using the SUPERCARBA medium and performing the Carba NP test on isolated colonies might be proposed as the recommended strategy for screening of carbapenemase producers [105].

In order to avoid the additional 24 h to 48 h before the carriage status of the patient can be established using those screening media an in-house quantitative real-time PCR assay using the TaqMan chemistry has been developed to detect the NDM-encoding genes directly from spiked stool samples. The bacterial extraction from stool samples was performed manually or adapted to a fully automated extraction system. This assay was found to be 100% specific and sensitive with detection limits reproducible below 1 × 10^1^ CFU/100 mg of feces [95]. However, this technology remains expensive and is thus considered to be a valuable tool in the follow-up of an outbreak and cohorting of colonized patients.

## 9. Conclusion

The rapid diffusion of NDM producers is of particular concern since this now corresponds to a worldwide-located outbreak. Additional knowledge in relation to the driven forces behind the spread of those multidrug-resistant isolates is now required, in order to better understand the dynamics of the NDM producers.

Among the most important features of NDM producers, one may retain that those NDM producers are not only nosocomial enterobacterial pathogens, but also community-acquired Enterobacteriaceae or other Gram-negative species, such as* A. baumannii*. Such diffusion pattern of multidrug resistance is unique for NDM producers and not observed currently for none of the producers of other types of carbapenemases (OXA-48, KPC, IMP…).

The reservoir of those NDM producers is mainly located in Southeast Asia where the rate of carriers is estimated to be ca. 20%. However, it is difficult to predict the number of tourists in India, Pakistan, and Bangladesh (more than 10 million in 2012) that will bring back those NDM producers at a carrier stage in stools. The size of that reservoir may explain the rapidity of the dissemination of NDM producers worldwide. Accordingly, NDM producers are now on the top list of carbapenemase producers in European countries such as the UK and even in France.

Due to the population demography of the NDM reservoirs coupled with the difficulties to implement hygiene measures or an efficient antibiotic stewardship program in those countries, the outbreak will not stop spontaneously. On the opposite, we will see an acceleration of the NDM producers spread worldwide. Hopefully the rate of NDM producers will not reach those currently observed for ESBL producers (20–80% worldwide). The spread of ESBL producers is an important driving force for usage of carbapenems that will enhance selection of carbapenemase producers. The only significant action we may actually take currently in Western countries which are not endemic for carbapenemase producers is to sustain the wide usage of rapid detection techniques now available and the extended screening of potential carriers at least in hospitals.

It is likely that novel antibiotic molecules such as the combination of aztreonam and avibactam may be launched in a near future and may bring some therapeutical openings. However there is an urgent need to finance novel research programs for discovering novel anti-Gram negatives molecules and to implement worldwide-located surveillance network of multidrug antibiotic resistance with sentinel labs.
